# Bioactive Chitin-Based Thermosensitive Hydrogel Reinforces Stem Cell Therapy for Osteoarthritis

**DOI:** 10.34133/bmr.0382

**Published:** 2026-06-23

**Authors:** Yiming Zhang, Xinbing Ren, Xiaoyu Duan, Xinyu Zhao, Yufeng Liu, Zicheng Dai, Weizhi Liu, Xiaohui Xu, Sudan Zhang, Guangmin Zhang, Xiaolei Dong, Yuping Yang, Jiane Liu, Yunfeng Gao, Daijie Wang, Chong Sun, Baoqin Han, Zheng Wang, Shaoqi Tian

**Affiliations:** ^1^ Department of Orthopedic Surgery, The Affiliated Hospital of Qingdao University, Qingdao, Shandong 266000, China.; ^2^ College of Integrated Traditional Chinese and Western Medicine, Jining Medical University, Jining, Shandong 272067, China.; ^3^ Department of Genetics and Cell Biology, Basic Medical College, Qingdao University, Qingdao, Shandong 266071, China.; ^4^ College of Marine Life Sciences, Ocean University of China, Shandong 266000, China.; ^5^ Department of Reproductive Medicine, The Affiliated Hospital of Qingdao University, Qingdao, Shandong 266000, China.; ^6^ Qingdao Anyrule Biological Health Technology Co., Ltd, Qingdao, Shandong 266114, China.; ^7^ International Joint Laboratory of Medicinal Food R&D and Health Products Creation/Biological Engineering Technology Innovation Center of Shandong Province, Heze Branch of Qilu University of Technology (Shandong Academy of Sciences), Heze, Shandong 274000, China.

## Abstract

Osteoarthritis (OA) is a progressive joint disorder that predominantly affects elderly and postmenopausal individuals. Current therapies offer only transient symptom relief and are associated with significant adverse effects. Mesenchymal stem cell (MSC) therapy holds promise for OA treatment, but challenges such as poor in vivo persistence and migration away from target sites hinder its clinical application. Here, we develop a bioactive, thermosensitive hydroxypropyl chitin (HPCT) hydrogel as an injectable platform to enhance MSC-based therapy. In both papain-induced early-stage and surgically induced late-stage OA models, intra-articular injections of MSCs combined with HPCT hydrogel significantly enhance therapeutic efficacy within the osteoarthritic joint environment. This bioactive, thermosensitive hydrogel is associated with attenuation of mechanical-stress-related ferroptotic signatures in chondrocytes through the establishment of a protective biomechanical microenvironment. MSCs embedded within the hydrogel adopt a spheroidal configuration, which improves their viability, enhances their anti-inflammatory properties, and prolongs their retention at the site of injury. These combined effects promote cartilage repair, regeneration, and sustained joint homeostasis. Mechanistically, these effects are accompanied by modulation of mechanotransduction-related pathways, including reduced Piezo1 expression and restoration of GPX4-associated antioxidant capacity. Our findings highlight HPCT-based tissue engineering as a promising therapeutic strategy for addressing OA pathophysiology and improving long-term clinical outcomes.

## Introduction

Osteoarthritis (OA) is a degenerative disease that disproportionately affects elderly and postmenopausal individuals, presenting a challenging set of problems [1]. Characterized by the progressive degradation of articular cartilage, OA leads to chronic pain, stiffness, and impaired joint function [2]. Despite its prevalence and the associated disability, their underlying molecular pathogenesis remains poorly understood, and the only available therapies consist of oral nonsteroidal anti-inflammatory drugs and analgesics, or intra-articular injections of corticosteroids or hyaluronic acid for advanced disease [3]. However, these treatments have long been contentious and controversial—they are primarily palliative, offering only short-term symptom relief without addressing the underlying disease mechanisms [4]. Moreover, significant adverse effects associated with these therapies limit their long-term efficacy in clinical applications [5].

A growing body of preclinical and clinical evidence has highlighted mesenchymal stem cell (MSC) therapy as a promising approach for OA treatment [6]. Implantation of MSCs, which are derived from the umbilical cord, umbilical cord blood, or adipose tissue [7], have demonstrated superiority over that of autologous chondrocytes, which tend to undergo dedifferentiation during in vitro expansion [8]. Mechanistically, MSCs injected into sites of inflammation and injury secrete bioactive factors that modulate the inflammatory environment, inhibit cartilage matrix degradation, and promote cartilage repair [9]. Notwithstanding these promising attributes, significant challenges remain in the clinical application of MSCs for OA, particularly their limited in vivo persistence and tendency to migrate away from target sites following intra-articular injection [10]. The present study was designed to develop an advanced tissue engineering system aiming to enhance the therapeutic potential of MSCs in OA treatment.

Using a combination of innovative techniques, we show that intra-articular injections of MSCs, when combined with a hydroxypropyl chitin (HPCT) hydrogel, a thermosensitive and bioactive material, significantly enhance therapeutic efficacy within the osteoarthritic joint. By mitigating mechanical-stress-induced chondrocyte ferroptosis, the HPCT hydrogel provides a supportive microenvironment that potentiates MSC-mediated therapeutic effects. Furthermore, MSCs embedded within the hydrogel adopt a spheroidal configuration, which not only improves their viability and anti-inflammatory potential but also prolongs their retention at the injury site, thereby maintaining chondrocyte homeostasis and promoting sustained cartilage repair and regeneration.

## Materials and Methods

### Synthesis and characterization of HPCT

The HPCT was prepared via a homogeneous hydroxypropylation reaction adapted from our established protocols [11,12]. In brief, purified chitin powder (molecular weight [Mw] = 255 kDa, degree of acetylation [DA] = 96.1%) was dispersed in sodium hydroxide/urea solution, frozen at −30 °C for 24 h, and transferred to a 3-necked flask equipped with a condenser. 1,2-Epoxypropane was added dropwise at 4 °C under continuous stirring for 24 h. The reaction mixture was neutralized with HCl, dialyzed against deionized water, and freeze dried to yield HPCT powder. HPCT was characterized by FT-IR and ^1^H NMR. Substitution degree of hydroxypropyl (DH) in HPCT was calculated according to the following formula [13]:

$$\mathrm{DH}\%=\frac{R_{C/N}-5.145-1.715\cdot \mathrm{DA}}{2.573}\times 100\%$$ (1)

HPCT was dissolved overnight at 4 °C in Dulbecco’s Modified Eagle’s Medium and Ham’s F-12 Nutrient Mixture (1:1) for cell-culture experiments and in physiological saline for animal experiments.

### Cell lines and cell culture

Human umbilical cord MSCs (SCSP-403) were maintained in UltraGRO-Advanced media (Helios, HPCFDCRL05) supplemented with 12.5% fetal bovine serum (Gibco, 10091-148) at 37 °C and 5% CO_2_. Cultures were passaged every 3 to 4 d by adding 0.25% trypsin (Gibco, 25200-056) for 5 to 10 min and re-plating at a 1:4 ratio.

### Cell Counting Kit-8 assay

Twenty microliters of Cell Counting Kit-8 (Beyotime, C0038) solution was added to each well and incubated at 37 °C for 2 h. Absorbance was measured at 450 nm using a full-wavelength microplate reader (Thermo Scientific, Multiskan GO). Cell viability from 3 independent experiments was calculated using Graph Pad Prism 8.0 [14].

### Experimental animals

Female Sprague–Dawley rats (8 wk old) were purchased from Beijing HFK Bioscience Co., Ltd. (Beijing, China). The weight of the rat is about 250 g, and the license number is SCXK2020-0005. All animals were housed in a specific pathogen-free facility under standard conditions (temperature: 25 ± 3 °C; humidity: 60% to 70%) with a 12:12-h light–dark cycle. All animal procedures were approved by the Institutional Animal Ethical Committee and Use Committee of Qingdao University (QDU-AEC-2024418), and all animal experiments follow the guidelines of the National Research Council Guide for the Care and Use of Laboratory Animals.

### OA rat model

Operation-induced OA model: rats were anesthetized, the knee joint area was shaved, and the skin was disinfected. A small incision was made at the knee joint using a scalpel, and blunt dissection was performed to expose the joint. Muscle tissue near the knee was retracted to expose the trochlear groove. The fat pad within the joint cavity was separated using micro forceps, and the knee joint was flexed to a 90° angle. The anterior cruciate ligament was transected with microsurgical scissors, and the medial meniscus was excised. The drawer test was conducted to confirm the instability of joint [15].

Papain-induced OA model: 0.2 ml of 4% papain was injected directly into the knee joint cavity on days 1, 4, and 7 to induce OA through enzymatic cartilage degradation [16].

### Scanning electron microscopy

The HPCT hydrogel was rapidly frozen in liquid nitrogen, freeze dried, carefully sectioned to expose cross-sectional surface, sprayed with a thin layer of gold, and imaged using scanning electron microscopy (JEOL, JSM-7800F) at 20 keV under vacuum.

### High-performance-gel-permeation-chromatography analysis

Mw distribution during degradation was analyzed using high-performance gel permeation chromatography with a Thermo U3000 system, BRT105-103-101 columns, and a refractive index detector. The standard curve (*y* = −0.2042*x* + 11.243, *R*^2^ = 0.9994) was used to calculate Mw.

### Rheological measurement

Rheological properties of HPCT hydrogels were analyzed with a rotary rheometer (TA Instruments DHR 10; Waters Technology Shanghai Limited). For dynamic temperature scans, HPCT hydrogel was measured from 10 to 40 °C at a rate of 2 °C/min with 1% constant strain and 1 rad/s constant frequency. The changes in the storage modulus (*G*′) and loss modulus (*G*″) were recorded. The phase-transition temperature was identified at the *G*′ and *G*″ intersection. For injectability tests, HPCT hydrogel was analyzed under shear rates ranging from 0.01 to 100 s^−1^ at 37 °C. For dynamic frequency scans, HPCT hydrogel was measured from 0.1 to 100 rad/s under 1% strain at 37 °C.

### Enzyme-linked immunosorbent assays

Samples and standards were diluted in assay buffer, incubated at room temperature for 2 h, incubated with primary antibody for 1 h, incubated with secondary antibody, washed 3 times, incubated with TMB substrate solution in the dark for 30 min, and stopped with 1 M sulfuric acid solution. Absorbance at 450 nm was recorded using a microplate reader [17,18]. The following enzyme-linked immunosorbent assay (ELISA) kits were used in ELISA experiments: Rat TNF alpha ELISA Kit (Elabscience, E-EL-R2856), Rat IL-1β ELISA Kit (Elabscience, E-EL-R0012), TSG-6 ELISA Kit (JONLNBIO, JL54324-96T), IL-10 ELISA Kit (JONLNBIO, JL13427-96T), TGF-β1 ELISA Kit (Elabscience, E-EL-0162), lipid peroxide ELISA Kit (JONLNBIO, JL21034), and malondialdehyde ELISA Kit (Elabscience, E-EL-0060).

### Glutathione assay

The synovial fluid glutathione (GSH) assay was performed according to the manufacturer’s protocol (Solarbio, BC1175). Twenty microliters of H_2_O or synovial fluid sample, 140 μl of Reagent 2, and 40 μl of Reagent 3 were added to a micro-glass cuvette and incubated for 2 min. The absorbance for blank (*A*_1_) and sample (*A*_2_) was recorded at 412 nm. Absorbance difference (Δ*A*) was calculated as Δ*A* = *A*_2_ − *A*_1_. Sample concentration was determined by substituting Δ*A* into the standard curve equation [19].

### Lipid peroxidation assay

The C11 lipid peroxidation assay was performed according to the instructions of the Beyo3D lipid peroxidation detection kit (Beyotime, S1111S). Cells were cultured in 6-well plates for 2 d, washed twice with phosphate-buffered saline (PBS), incubated with 1 ml working solution for 30 min in the dark, washed with PBS, and imaged under a fluorescence microscope. Fluorescence intensity was quantified using ImageJ.

### Immunoblotting

Cells were lysed in RIPA buffer (Beyotime, P0013B) containing protease and phosphatase inhibitors. Protein concentrations were measured using a BCA Protein Assay Kit (Beyotime, P0012). Forty micrograms of total protein was separated on SDS–PAGE gels and transferred to PVDF membranes (Millipore). Membranes were blocked with 5% nonfat milk in TBST for 1 h at room temperature, incubated with primary antibodies overnight at 4 °C, washed, and incubated with secondary antibodies for 1 h at room temperature. Protein bands were visualized using enhanced chemiluminescence (Elabscience) and quantified with the OI600 imaging system (Guangyi, China). Antibodies (dilutions) used for Western blotting were GAPDH (1:4,000), Piezo1 (1:2,000), P65 (1:2,000), GPX4 (1:2,000), and TLR2 (1:2,000).

### Flow cytometry

Cells were dissociated into single-cell suspension, blocked in PBS with 5% of newborn calf serum, and incubated with fluorescently labeled CD44, CD90, and CD105 antibodies (5 μl each) for 20 min on ice in the dark. Cells were washed, filtered, and analyzed on a BD LSRII instrument [20].

### Synovial fluid viscosity measurement

Synovial fluid from 3 rats was combined and diluted with equal volume of PBS. The viscosity measurement was performed at 37 °C with shear rates ranging from 0.01 to 100 s^−1^ using a rotational rheometer (TA Instruments DHR 10; Waters Technology Shanghai Limited).

### RNA isolation

Total RNA was isolated using FreeZol Reagent (Vazyme, R401-01), treated with DNase I (Yeasen, 10611ES76), precipitated, washed with 70% ethanol, and dissolved in H_2_O.

### Reverse transcription and quantitative polymerase chain reaction

One microgram of total RNA was reverse transcribed using random hexamers and Hiscript III Reverse Transcriptase (Vazyme, R302-01). Then, 20 ng cDNA was used in each reverse transcription–quantitative polymerase chain reaction (RT-qPCR) on a CFX96 instrument using Taq Pro Universal SYBR qPCR Master Mix (Vazyme, Q311-02). Standard curves were generated for each primer set to calculate Ct values with the expression threshold set to 100 RFU. All expression values were scaled to GAPDH. The gene-specific primers used in all PCR-based analyses are listed in Table 1.

**Table 1.** All gene-specific primers in polymerase-chain-reaction-based analyses

TGFβ1-F	5′-CTGCAAGACTATCGACATGG-3′
TGFβ1-R	5′-AGATAACCACTCTGGCGAGT-3′
IL-10-F	5′-ATGCCCCAAGCTGAGAACCAAGACCCA-3′
IL-10-R	5′-TCTCAAGGGGCTGGGTCAGCTATCCCA-3′
TSG-6-F	5′-GGAATTCATGATCATCTTAATTTACT-3′
TSG-6-R	5′-CGGGATCCTAAGTGGCTAAATCTTCC-3′
CD105-F	5′-CCACTAGCCAGGTCTCGAAG-3′
CD105-R	5′-GATGCAGGAAGACACTGCTG-3′
CD90-F	5′-ATGAACCTGGCCATCAGCA-3′
CD90-R	5′-GTGTGCTCAGGCACCCC-3′
CD44-F	5′-GACACTATTGCTTCAATGCTTCAGC-3′
CD44-R	5′-GATGCCAAGATGATCAGCCATTCTGGAAT-3′

### RNA-sequencing analysis

RNA libraries were prepared using the VAHTS Universal V8 RNA-Seq Library Prep Kit (Vazyme, NRM605-01) and sequenced on the MGI-SEQ 2000 platform (BGI Technology). Demultiplexed reads were mapped to the Hg19 human genome with HISAT2 and analyzed with DESeq2 for differential expression. Mapped reads were reconstructed using Stringtie. For transcript quantification and differential expression, demultiplexed reads were analyzed with RSEM. Differential expression for all groups was determined using DESeq2. Gene Ontology and KEGG pathway enrichment analysis were performed using WebGestalt (https://www.webgestalt.org/) [21].

### Joints scoring

Joint surfaces were evaluated for inflammation, cartilage integrity, and bone exposure based on a 0–4 scoring system: Score 0: smooth, light blue, and translucent cartilage surface; Score 1: smooth and intact cartilage surface with a slightly softer texture; Score 2: localized thinning with mild fiber bundle formation in some areas; Score 3: moderate fiber bundle formation on the cartilage surface; and Score 4: severe fiber bundle formation, visible cartilage defects, exposure of subchondral bone, and signs of bone sclerosis [22].

### Hematoxylin–eosin and Safranin O/Fast Green staining

Cartilage tissue was fixed in 4% paraformaldehyde at room temperature, embedded in paraffin, and cut into ultrathin sections (5 μm), followed by gradient dewaxing. Sections were stained with hematoxylin and eosin (H&E; Beyotime, C0105S) or Safranin O/Fast Green (Solarbio, G1371), dehydrated, mounted on slides, and imaged by a light microscope (Nikon, Ni-U) [23].

### Immunohistochemistry

Paraffin-embedded sections were subjected to antigen retrieval with 0.01 M sodium citrate buffer (pH 6.0) at 90 °C, blocked with 5% bovine serum albumin, incubated with primary antibody overnight at 4 °C, washed, incubated with secondary antibody at 37 °C for 1 h, washed with PBS, stained with diaminobenzidine, and imaged by light microscope (Nikon, Ni-U). The collagen II antibody (Immunoway, YT1022) was used for immunohistochemistry experiments [24].

### Immunofluorescence staining

Cells were fixed in 4% paraformaldehyde for 10 min, permeabilized with PBS containing 0.5% Triton X-100, blocked with 5% goat serum for 1 h, incubated with primary antibodies at room temperature for 2 h, washed, incubated with secondary antibody for 1 h at room temperature, coverslipped, and imaged on a confocal microscope (Leica, TCS SP5). The following antibodies were used in immunofluorescence staining experiments: matrix metalloproteinase 13 (MMP13; Immunoway, YT279), GPX4 (Servicebio, GB114327), NF-κB (Servicebio, GB11997), and TLR2 (Servicebio, GB113378) [25].

### Reactive-oxygen-species detection

Frozen tissue sections were rewarmed to room temperature, marked, quenched, and washed for 10 min. Marked area was incubated with reactive oxygen species detection dye (Sigma-Aldrich, D7008) at 37 °C in a darkened incubator for 30 min, washed 3 times, incubated with DAPI (Servicebio, G1012), washed, and imaged under fluorescence microscopy [26].

### Magnetic resonance imaging

MRI scans were obtained using a 3.0-T Philips scanner (GE Discovery MR750). Cartilage thickness was quantified by selecting 3 regions of interest (ROI): the thickest mid-layer of the cartilage and one layer anterior and posterior to this site. For each ROI, the distance between the cartilage surface and the subchondral bone was measured, and the average value was calculated. Signal intensity of the cartilage was measured at the thickest region in each ROI across 3 consecutive layers, with the mean value recorded to ensure accuracy. To reduce measurement error, the signal-to-noise ratio was calculated for each region, allowing for standardized signal-intensity assessments.

Articular cartilage injury was graded based on the International Cartilage Regeneration and Joint Preservation Society classification system [27]: Grade 0: intact cartilage surface contour with normal thickness; Grade 1: loss of cartilage layered structure with slight surface irregularity; Grade 2: moderately irregular cartilage surface with defect depth <50% of the full thickness; Grade 3: severely irregular cartilage surface with defect depth >50% of the full thickness but not completely detached; and Grade 4: complete cartilage loss with detachment, exposing the subchondral bone.

### Live imaging

MSCs genetically labeled with firefly luciferase were injected into the experimental rats. D-luciferin sodium salt was administered via injection. Rats were anesthetized and placed on the imaging platform inside the imaging darkbox. Both bright-field and dark-field imaging were conducted to capture the bioluminescent signal. The signals were analyzed using Living Image software 4.4 for IVIS spectrum and imaged under a live animal imaging device (IVIS Lumina XRMS III) [28].

### Quantification and statistical analysis

All assays were repeated 3 times. Statistical analyses were performed using GraphPad Prism 9 software. Data are represented as mean ± standard error of the mean. One-way analysis of variance was used to compare differences among multiple groups. Pairwise comparisons between groups were performed using the Student *t* test. A *P* value of less than 0.05 (*P* < 0.05) was considered statistically significant.

## Results

### Characterization of the HPCT hydrogel

**Fig. 1.**
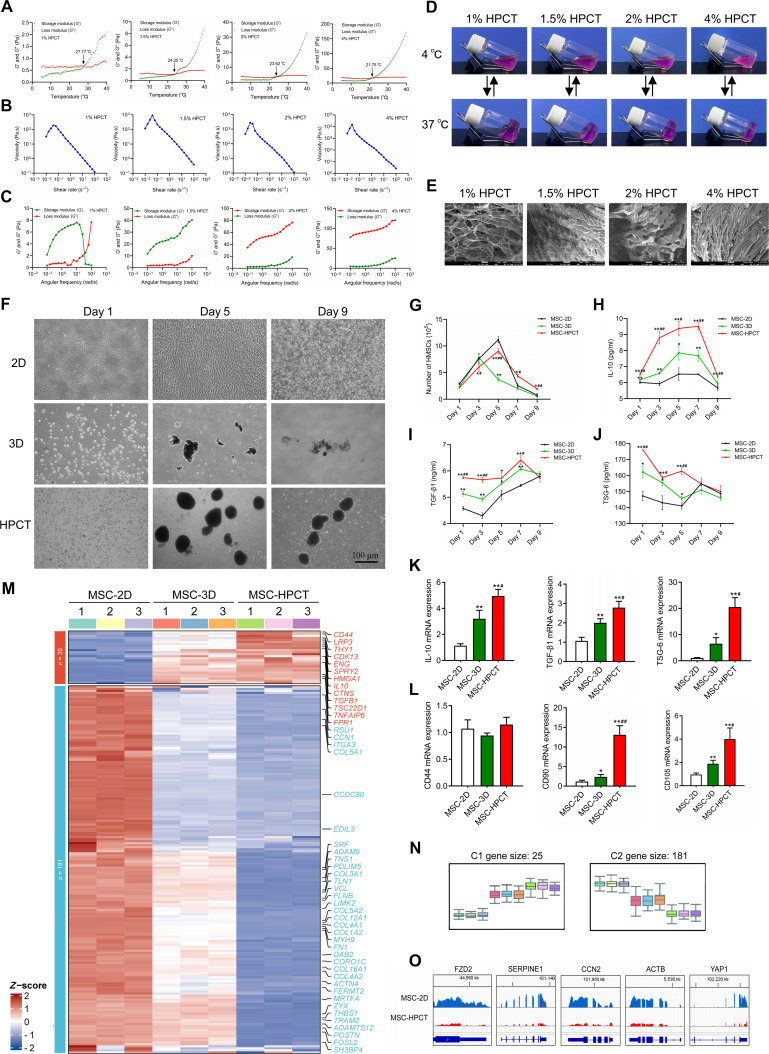
Hydroxypropyl chitin (HPCT) hydrogel provides a thermosensitive 3-dimensional (3D) microenvironment that enhances mesenchymal stem cell (MSC) self-renewal and immunomodulatory function. (A) Temperature transition points of hydrogels at varying concentrations were measured by dynamic temperature scanning. (B) Viscosity of HPCT hydrogels was measured under varying shear rates. (C) Storage modulus (*G*′) and loss modulus (*G*″) variations of hydrogels were measured at different concentrations under varying angular velocities. (D) Morphological analysis of HPCT hydrogels at 4 and 37 °C. (E) Microstructure of HPCT hydrogels analyzed under scanning electron microscopy. (F) Morphological analysis at day 1, day 5, and day 9 MSCs cultured at conventional 2-dimensional (2D), 3D, or embedded in HPCT hydrogel. Scale bar: 100 μm. (G to J) Cell counts (G) and enzyme-linked immunosorbent assay (ELISA) analysis of IL-10 (H), TGF-β1 (I), and TSG-6 (J) during a 9-d culture time course. (K and L) Quantitative polymerase chain reaction (qPCR) analysis of IL-10, TGF-β1, and TSG-6 (K) and CD44, CD90, and CD105 (L) in MSCs cultured at conventional 2D, 3D, or embedded in HPCT hydrogel. (M) Heatmap representation of differentially expressed genes (up-regulated and down-regulated) across 2D, conventional 3D, and HPCT hydrogel conditions. (N) Boxplots of log2-transcripts per kilobase million values (RNA sequencing [RNA-seq]) in MSC-2D, MSC-3D, and MSC-3D HPCT. (O) Genome browser tracks showing RNA-seq signal at Hippo pathway-associated genes, comparing MSCs cultured in 2D and embedded in HPCT hydrogel. HMSCs, human mesenchymal stem cells. *P < 0.05, **P < 0.01, #*P* < 0.05, ##*P* < 0.01.

To address the limited in vivo persistence of MSCs following intra-articular injection for OA treatment, we developed an injectable, thermosensitive hydrogel based on HPCT (Fig. S1A), considering that chitin can degrade in vivo into glucosamine (GS) or *N*-acetyl-α-d-glucosamine (NAG), which are cartilage precursors. The synthesized HPCT (degree of hydroxypropyl substitution: 78.1%) demonstrated excellent water solubility at concentrations below 4%. Dynamic temperature scanning analysis revealed phase-transition temperatures of 27.77, 24.25, 23.62, and 21.75 °C for 1%, 1.5%, 2%, and 4% HPCT hydrogel, respectively, based on the intersection of the storage modulus (*G*′) and loss modulus (*G*″) (Fig. 1A). Viscosity analysis confirmed the shear-thinning behavior of the hydrogels. The peak viscosity of the 1% hydrogel did not exceed 10^3^ Pa·s, whereas the 1.5% and 2% hydrogels exhibited peak viscosities exceeding 10^3^ Pa·s, and the 4% hydrogel reached peak viscosities exceeding 10^4^ Pa·s (Fig. 1B). Dynamic mechanical analysis demonstrated that the 1.5% and 2% hydrogels exhibited more stable modulus profiles across varying angular velocities compared to others (Fig. 1C). All concentrations exhibited thermosensitive gelation at 37 °C, with higher concentrations showing faster gelation times (Fig. 1D). Scanning electron microscopy revealed that the 1%, 1.5%, and 2% hydrogels featured uniform, well-defined porous structures (20 to 80 μm), whereas the 4% hydrogel exhibited irregular and indistinct pores (Fig. 1E). Collectively, the 1.5% and 2% hydrogels were identified as the optimal candidates based on their thermosensitive properties, mechanical stability, and structural integrity.

### HPCT hydrogel promotes MSC self-renewal and immunomodulatory capacity

To evaluate the ability of HPCT hydrogel to support MSCs in 3-dimensional (3D) culture, we embedded MSCs in the 1.5% HPCT hydrogel. Compared to 2-dimensional (2D) culture and conventional 3D culture on ultra-low attachment plates, MSCs embedded in HPCT hydrogel exhibited significantly prolonged viability and maintained their spheroidal structures for up to 9 d (Fig. 1F and G). H&E-staining analysis revealed that, compared with the 2D group, MSCs in 3D culture formed spheroid-like aggregates with increased cell size. Furthermore, compared with the conventional 3D culture group, the HPCT hydrogel group exhibited more compact spheroid formation, increased spheroid numbers, and no obvious abnormal changes in cell morphology (Fig. S1B). The stationary culture further enhanced spheroid formation and cell renewal in the presence of Rho-associated protein kinase inhibitors (Fig. S1C to G). Intriguingly, ELISA and qPCR analysis revealed increased secretion of anti-inflammatory cytokines IL-10, TGF-β1, and TSG-6 and elevated expression of stemness markers CD44, CD90, and CD105 compared to 2D and conventional 3D culture (Fig. 1H to L). Flow cytometry analysis of these CD markers across all groups yielded results consistent with the qPCR data (Fig. S1H to K).

To obtain molecular insights into these observed characteristics, we performed RNA-sequencing (RNA-seq) analysis on these cells. We reasoned that like anti-inflammatory cytokines and stemness markers, other genes associated with enhanced MSC identity are either up-regulated or down-regulated across 2D, conventional 3D, and hydrogel-embedded culture systems. RNA-seq analyses of MSCs in these culture systems identified 25 up-regulated genes and 181 down-regulated genes (Fig. 1M and N). The up-regulated gene group was enriched for gene ontologies associated with negative regulation of apoptotic process and T-cell proliferation, inflammatory response, and positive regulation of PI3K/Akt, ERK1/2, SMAD, and EGFR signaling (Fig. S2A). Additionally, heterotypic cell–cell adhesion was prominently represented. In contrast, the down-regulated genes were associated with particularly cell matrix adhesion, extracellular matrix (ECM) organization, and actin cytoskeleton organization, suggesting the involvement of mechanotransduction in MSC maintenance (Fig. S2B). This was further confirmed by the decreased expression of key Hippo pathway regulators (Fig. S2C), including Frizzled receptor 2 (FZD2), Serpin family E member 1 (SERPINE1), cellular communication network factor 2 (CCN2), Actin Beta (ACTB), and Yes1 associated transcriptional regulator (YAP1) (Fig. 1O), specifically in HPCT hydrogel-embedded MSCs. We further compared the effects of 1.5% HPCT and 4% HPCT on stemness and YAP expression. The results showed that the soft matrix reduced YAP expression while enhancing stemness, whereas the stiff matrix increased YAP expression and reduced stemness (Fig. S3A to D).

To test whether HPCT may gradually degrade within the joint cavity over time, we performed high-performance-gel-permeation-chromatography analysis of synovial fluid collected from the joint cavity at day 0 and day 9 after HPCT injection. As expected, at day 0, no detectable signal was observed in the chromatogram, indicating strong insolubility of HPCT. In contrast, at day 9, a symmetric and single peak appeared at a retention time of 42.178 min, with an average Mw of 427 Da, indicating that HPCT had degraded into oligosaccharides or monosaccharides (Fig. S4A). Given the established cellular bioactivity of GS and its derivative [29], we assessed contribution of GS, NAG, and their oligomeric derivatives chitosan oligosaccharides and chitin oligosaccharides (CTOS) on MSC performance. While not significantly increasing the MSC numbers, NAG and CTOS showed markedly enhanced self-renewal effects without altering cell morphology (Fig. S4B and C). Importantly, both NAG and CTOS promoted the secretion of IL-10, TGF-β1, and TSG-6, with CTOS exhibiting stronger effects on IL-10 and TSG-6, and NAG preferentially enhancing TGF-β1. Notably, CTOS significantly increased CD105 expression, while both NAG and CTOS up-regulated CD90 (Fig. S4D to I).

These findings demonstrate that HPCT hydrogel not only provides a supportive 3D microenvironment for MSC maintenance but also enhances their self-renewal and immunomodulatory potential.

### HPCT hydrogel prolongs MSC retention in the joint cavity

**Fig. 2.**
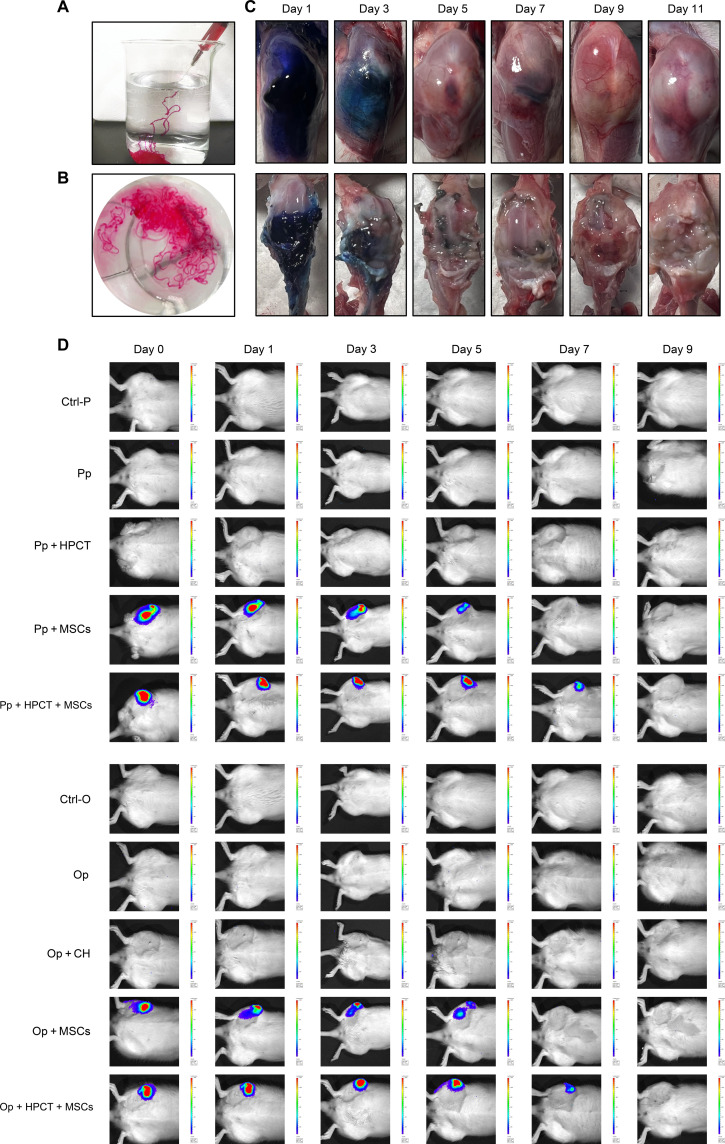
HPCT hydrogel enabled long-term in vivo survival and retention of MSCs in joint cavity. (A and B) Morphology of HPCT hydrogel stained with phenol red immediately after injection into warm water (A) and 30 min postinjection (B). (C) Toluidine blue staining of HPCT hydrogel in rat articular cavity at day 1, day 3, day 5, day 7, day 9, and day 11 postinjection. (D) Fluorescence images of luciferase-positive MSCs in rat articular cavity at day 0, day 1, day 3, day 5, day 7, and day 9 postinjection. Op, operation; Pp, papain. *n* = 8 per group.

To examine the injectability and stability of HPCT hydrogel, we performed a series of in vitro and in vivo studies. In vitro assessments demonstrated that the hydrogel retained structural integrity and shape fidelity in aqueous conditions at 37 °C for at least 30 min postinjection (Fig. 2A and B). Correspondingly, intra-articular injections of HPCT hydrogel into rat joint cavities demonstrated stability for up to 9 d, with no evidence of adverse inflammatory responses, as verified by histological analysis (Fig. 2C and Fig. S5A to D).

To further test the consequence of the addition of HPCT hydrogel in MSC retention within rat joint cavities, we employed bioluminescent imaging of luciferase-expressing MSCs in both papain- and operation-induced OA rat models. MSCs co-delivered with HPCT hydrogel exhibited significantly prolonged retention within the joint cavity (Fig. S5A). Fluorescence intensity indicated a 1.5-fold increase in MSC localization, extending retention from 5 to 9 d compared to MSCs injected alone (Fig. 2D and Fig. S6B).

These in vitro and in vivo data highlight the capacity of HPCT hydrogel to enhance MSC retention and stability, addressing a critical limitation of MSC-based OA therapy.

### HPCT hydrogel/MSC synergy facilitates cartilage regeneration

**Fig. 3.**
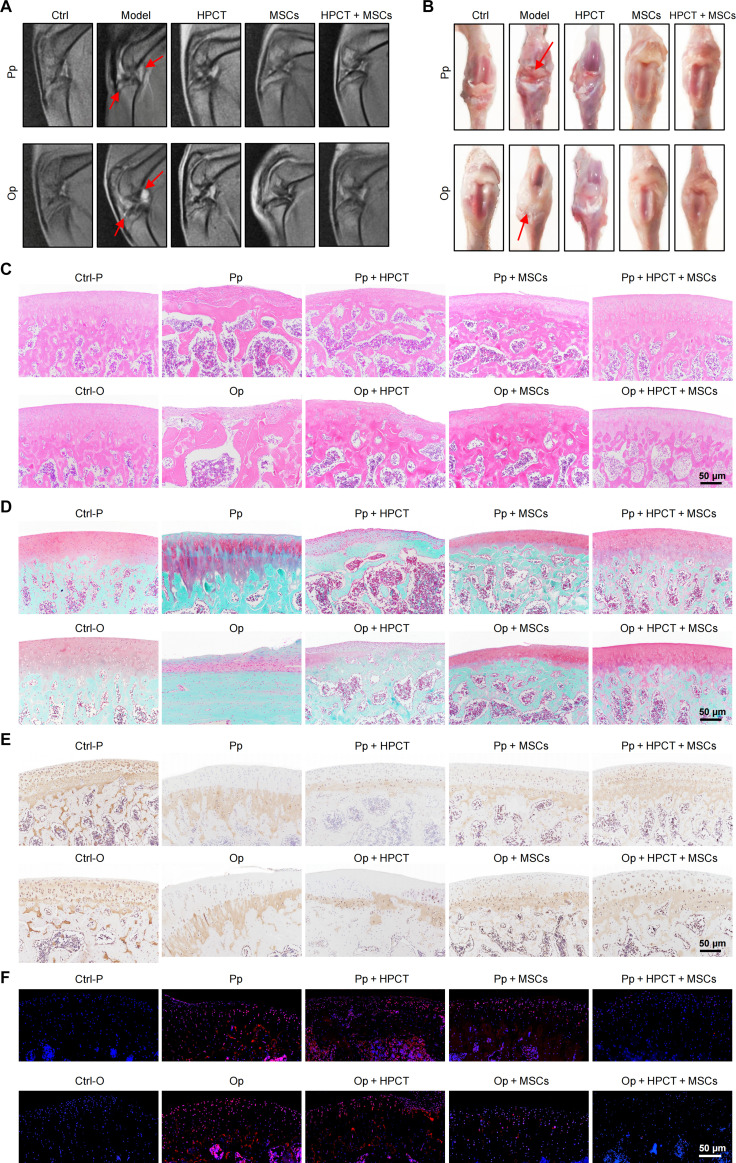
HPCT hydrogel/MSC synergy facilitates cartilage regeneration in operative- and papain-induced osteoarthritis (OA) model. (A to F) Representative image of MRI (A), gross anatomical (B), hematoxylin and eosin (H&E) staining (C), Safranin O/Fast Green staining (D), immunohistochemistry staining of collagen II (E), and immunofluorescence staining of matrix metalloproteinase 13 (F) in rat articular cartilage at day 9 posttreatment. *n* = 8 per group.

We next investigate the therapeutic potential of HPCT hydrogel combined with MSCs for cartilage repair. MRI analyses demonstrated that the combined therapy markedly reduced joint effusion and osteophyte formations compared to MSCs alone or untreated controls (Fig. 3A). Quantitative scoring based on the International Cartilage Regeneration and Joint Preservation Society and bone and joint criteria corroborated these observations: While individual treatments with HPCT hydrogel or MSCs achieved approximately 20% reductions in pathological scores, the combined HPCT hydrogel/MSC therapy achieved reductions of up to 50%, highlighting the superior efficacy of the HPCT/MSCs combined therapy (Fig. S7A).

Macroscopic examination of dissected joints in the OA models revealed extensive cartilage hyperplasia and inflammatory responses. Both MSC therapy alone and HPCT/MSCs combined therapy alleviated these pathological changes, with the latter yielding more pronounced improvements, including reduced inflammatory infiltration and smoother cartilage surfaces (Fig. 3B and C and Fig. S7B to D). Safranin O/Fast Green staining indicated a significant increase in the glycosaminoglycan-to-bone ratio in the cartilage of treated joints, with the highest levels observed in the combined therapy group, underscoring its enhanced regenerative effect (Fig. 3D and Fig. S7E). Immunohistochemical analyses further confirmed the regenerative capacity of the combined therapy. Collagen II, a critical cartilage matrix protein, was significantly restored in both treatment groups, with the highest levels in the combined therapy cohort (Fig. 3E and Fig. S7F). Conversely, MMP13, a marker of cartilage degradation, was profoundly suppressed by the HPCT hydrogel/MSC therapy (Fig. 3F and Fig. S7G). Importantly, systemic safety evaluations across treatment groups revealed no toxicity or adverse effects, confirming the biocompatibility of the combined therapy (Fig. S8A to L).

Together, our scoring, histology, and immunohistochemistry data highlight the synergistic effects of HPCT hydrogel and MSCs in enhancing cartilage regeneration. The therapy promotes glycosaminoglycan synthesis, collagen II production, and cartilage thickening while simultaneously suppressing cartilage degradation.

### HPCT hydrogel/MSC therapy mitigates inflammation in articular cartilage

**Fig. 4.**
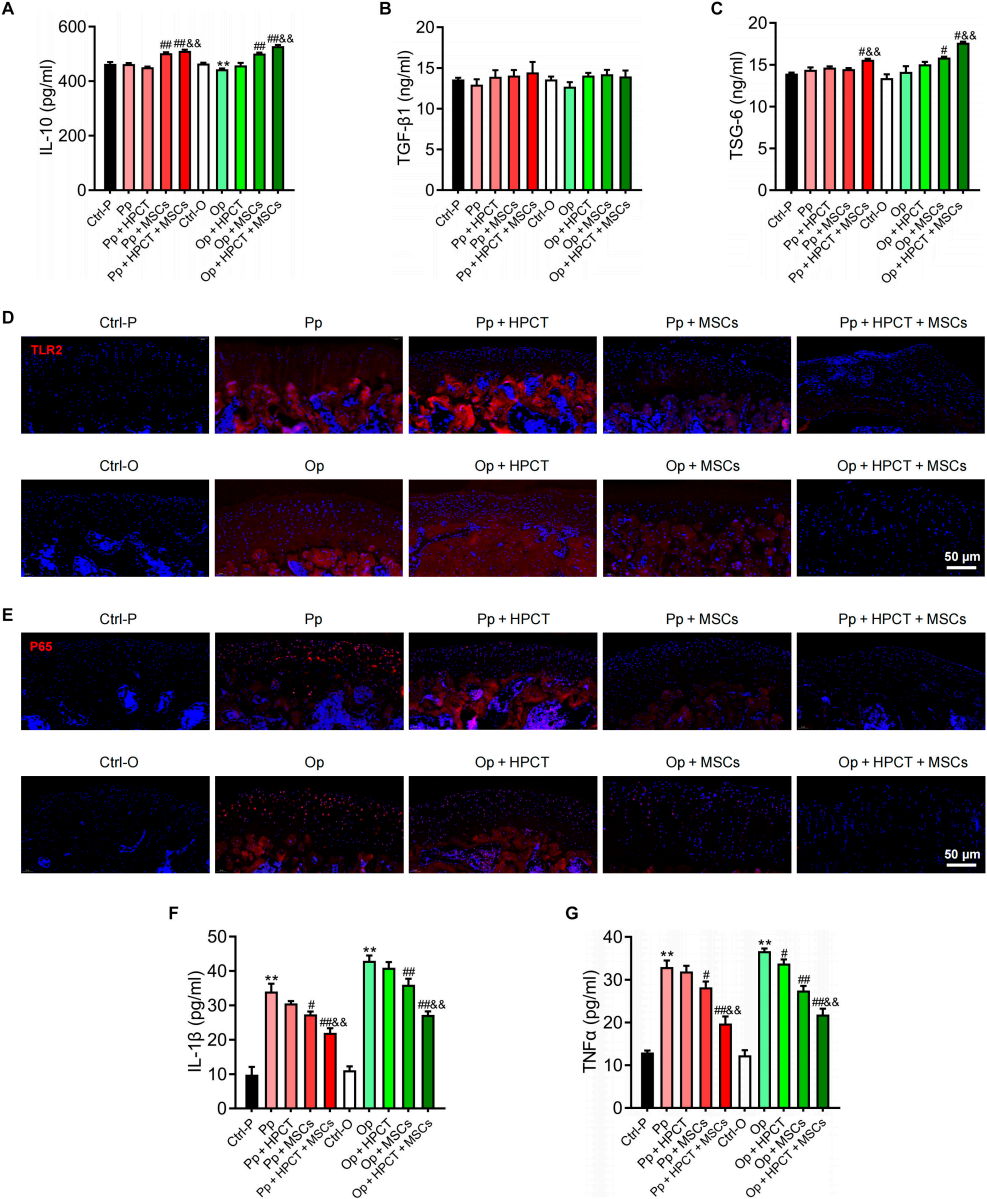
HPCT hydrogel/MSC therapy mitigates inflammation in articular cartilage in operative- and papain-induced OA model. (A to C) ELISA analysis of IL-10 (A), TGF-β1 (B), and TSG-6 (C) in synovial fluid at day 9 posttreatment. (D and E) Representative immunofluorescence images of TLR2 (D) and P65 (E) in rat articular cartilage at day 9 posttreatment. (F and G) ELISA analysis of IL-1β and TNF-α in synovial fluid at day 9 posttreatment. *n* = 8 per group. **P < 0.05, #*P* < 0.05, ##*P* < 0.01, &&*P* < 0.01.

Since the data presented above implicate the synergistic effect of HPCT combined with MSCs in cartilage repair, we assessed their effects on inflammation, a pivotal driver of OA progression. ELISA of joint fluid samples revealed elevated anti-inflammatory factors TSG-6 and IL-10 in the combined HPCT/MSC therapy group compared to all other groups, in both papain- and operation-induced OA models (Fig. 4A and C). Interestingly, no significant differences were observed in TGF-β1 levels across all groups, indicating that TGF-β1 may play a limited role in the observed anti-inflammatory effects (Fig. 4B). To further validate the anti-inflammatory effects of HPCT/MSCs in vitro, chondrocytes were first treated with TNF-α (50 ng/ml) for 3 d to establish an inflammatory microenvironment model. Subsequently, HPCT–MSC spheroids were co-cultured with the inflamed chondrocytes. HPCT–MSC treatment restored the reduced cell number and abnormal morphology of chondrocytes under inflammatory conditions (Fig. S9A and B) and significantly suppressed the activation of the TLR2/NF-κB signaling pathway (Fig. S9C to E).

Immunofluorescence staining of joint tissues showed elevated expression of the pro-inflammatory pathway proteins P65 and TLR2 following OA induction. Treatment with MSCs alone attenuated this up-regulation, while the combined HPCT/MSC therapy exhibited significantly greater reductions in the expression of both proteins similar to that of control group (Fig. 4D and E and Fig. S9F and G). Similarly, ELISA analysis of pro-inflammatory cytokines in joint fluid demonstrated that levels of IL-1β and TNF-α were significantly reduced by MSC therapy, with the combined HPCT/MSC therapy producing the most pronounced decreases (Fig. 4F and G). Notably, HPCT hydrogel alone did not achieve these effects, highlighting the necessity of MSCs in mediating the anti-inflammatory response.

These data demonstrate enhanced dual regulation of pre- and pro-inflammatory factors of the MSC therapy in the presence of HPCT hydrogel in halting OA progression.

### HPCT hydrogel/MSC therapy alleviates chondrocyte ferroptosis

**Fig. 5.**
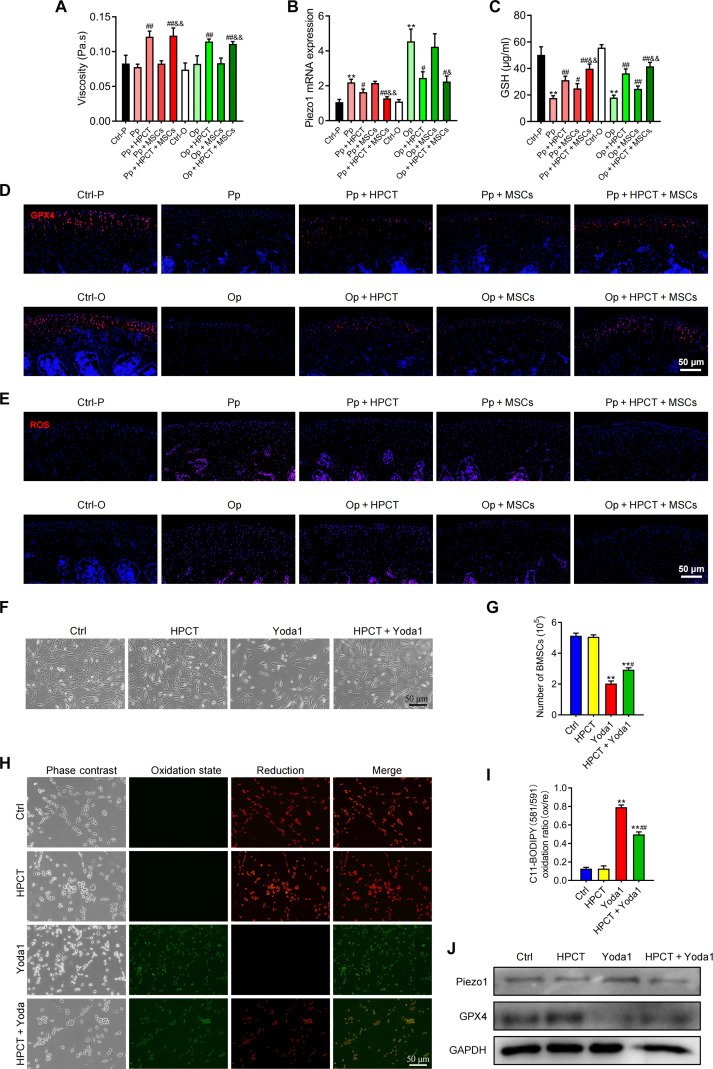
HPCT hydrogel/MSC therapy alleviates chondrocyte ferroptosis in operative- and papain-induced OA model. (A) Rheological analysis of viscosity in synovial fluid at day 9 posttreatment. (B) qPCR analysis of Piezo1 gene expression in rat articular cartilage at day 9. (C) Biochemical analysis of glutathione (GSH) in synovial fluid at day 9. (D and E) Representative immunofluorescence images stained GPX4 (D) and reactive oxygen species (E) in rat articular cartilage at day 9. *n* = 8 per group. (F and G) Representative images of cell morphology (F) and viability (G) in in vitro chondrocyte ferroptosis model induced by Yoda1 treatment for 2 d. (H and I) C11-BODIPY staining of chondrocytes (H), with quantitative analysis of lipid peroxidation based on the red/green fluorescence ratio (I). Western blot was used to detect the expression levels of Piezo1 and GPX4 proteins in each group in H (J). BMSCs, bone mesenchymal stem cells. **P < 0.05, #*P* < 0.05, ##*P* < 0.01, &*P* < 0.05, &&*P* < 0.01.

Considering the thermosensitive gelation features of HPCT hydrogel, we examined mechanical stress within the joint cavity among all groups. Measurement of joint viscosity revealed that HPCT hydrogel, either alone or in combination with MSCs, significantly enhanced joint viscosity compared to untreated controls, suggesting improved biomechanical support (Fig. 5A). Furthermore, the expression of Piezo1, a mechanosensitive ion channel, was markedly reduced following HPCT hydrogel administration. This reduction was more pronounced in the operation-induced OA model and further amplified by the addition of MSCs (Fig. 5B).

Studies in OA patient samples have indicated that Piezo1 is closely linked to ferroptosis, a newly discovered iron-dependent form of regulated cell death involved in OA occurrence and progression [30]. To this end, we evaluated key ferroptosis markers to assess the protective effects of these therapies. Levels of GSH, a critical antioxidant, were significantly elevated in joints treated with the HPCT hydrogel, with even greater increases observed in the HPCT/MSCs combination therapy group (Fig. 5C). Immunofluorescence analysis demonstrated substantial up-regulation of GPX4, another pivotal marker of ferroptosis, and a concurrent reduction in reactive oxygen species levels after HPCT treatment. These effects were notably more robust in the combined therapy group (Fig. 5D and E and Fig. S9H and I). Markers of lipid peroxidation, another hallmark of ferroptosis, were also significantly reduced. Levels of lipid peroxide products and malondialdehyde were markedly lower in joints treated with HPCT hydrogel, with the most profound reductions observed in the combination therapy group (Fig. S9J and K).

To further validate these findings in vitro, we established a chondrocyte ferroptosis model using Yoda1, a ferroptosis inducer, to stimulate cells for 2 d. Yoda1 treatment led to a significant reduction in cell number and induced irregular cellular morphology. In contrast, HPCT-embedded chondrocyte cells markedly restored both cell viability and morphological integrity (Fig. 5F and G). C11 staining validated these results, demonstrating that HPCT embedding treatment significantly reduced lipid peroxidation levels in chondrocytes (Fig. 5H and I). Consistently, western blot analysis revealed decreased Piezo1 protein expression while markedly increasing GPX4 levels (Fig. 5J and Fig. S9L and M), further supporting the role of HPCT in suppressing ferroptosis.

These results suggest that HPCT hydrogel treatment is associated with attenuation of chondrocyte ferroptosis by modulating mechanical signals. Moreover, the presence of MSCs further enhances these protective effects against cartilage degeneration and contributing to the restoration of joint homeostasis in OA.

### Long-term application of HPCT hydrogel/MSC therapy reverses OA hallmarks

**Fig. 6.**
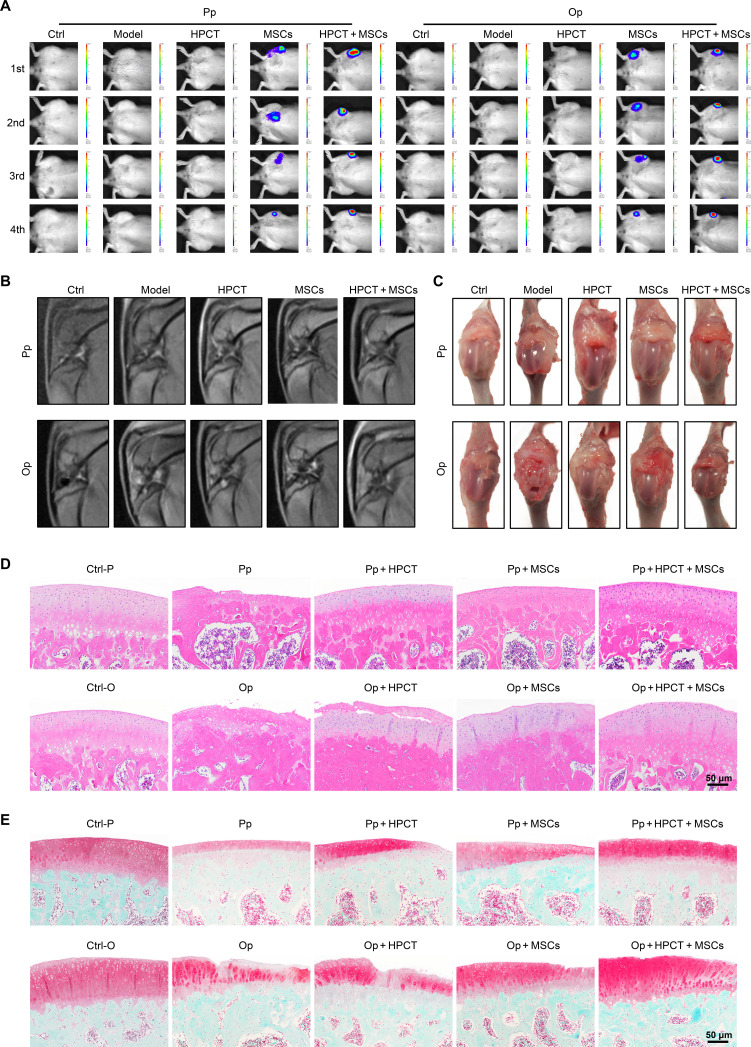
Long-term application of HPCT hydrogel/MSC therapy reverses OA hallmarks in operative- and papain-induced OA model. (A) Fluorescence images of MSCs in the articular cavity at day 4 posttreatment. (B to E) Representative image of MRI (B), gross anatomical (C), H&E staining (D), and Safranin O/Fast Green staining (E) at 36 d posttreatment. *n* = 8 per group.

To evaluate the sustained efficacy of HPCT hydrogel/MSC therapy in addressing cartilage damage, we implemented a regimen of multiple administrations at 9-d intervals. Treatments were initiated on day 29 after OA induction, followed by subsequent injections on days 38, 47, and 56, with MSC retention monitored via in vivo imaging 4 d posttreatment. Similar to imaging data presented above (Fig. S10A), MSC retention and aggregation within the joint cavity were significantly enhanced in the HPCT hydrogel group compared to MSCs alone, which exhibited substantial cell migration (Fig. 6A and Fig. S10B). MRI analysis revealed that long-term OA modeling caused severe joint effusion and osteophyte formation. The combination therapy markedly reduced both effusion and osteophyte severity compared to untreated controls and MSCs-alone groups (Fig. 6B), as shown by superior OA scores (Fig. S10C). Macroscopic examination further supported these findings, demonstrating pronounced reductions in fat hyperplasia and inflammatory damage in treatment groups, with the most significant improvements in the combined therapy group (Fig. 6C and Fig. S10D). Histological analyses confirmed the macroscopic outcomes: smoother cartilage surfaces and reduced inflammatory cell infiltration in MSC-treated groups, with the combined therapy exhibiting the most substantial improvements, as shown by H&E staining (Fig. 6D and Fig. S10E and F), and significantly increased cartilage polysaccharide content in the combined treatment group, as shown by Safranin O/Fast Green staining (Fig. 6E and Fig. S10G), highlighting enhanced cartilage regeneration. At the same time, levels of IL-10 and TSG-6 were significantly elevated in the combined therapy group, while pro-inflammatory cytokines IL-1β and TNF-α were markedly reduced (Fig. S11A to E). Consistent with previous findings, TGF-β1 levels remained unchanged across all groups (Fig. S11C). Additionally, ferroptosis-related marker GSH was significantly increased in the HPCT hydrogel group, with the combined treatment demonstrating the most pronounced effect (Fig. S11F). Long-term safety evaluations, including blood tests, liver function assays, and thymus and spleen indices, confirmed the excellent biocompatibility of the combined therapy, with all values remaining within normal physiological ranges (Fig. S12A to L).

**Fig. 7.**
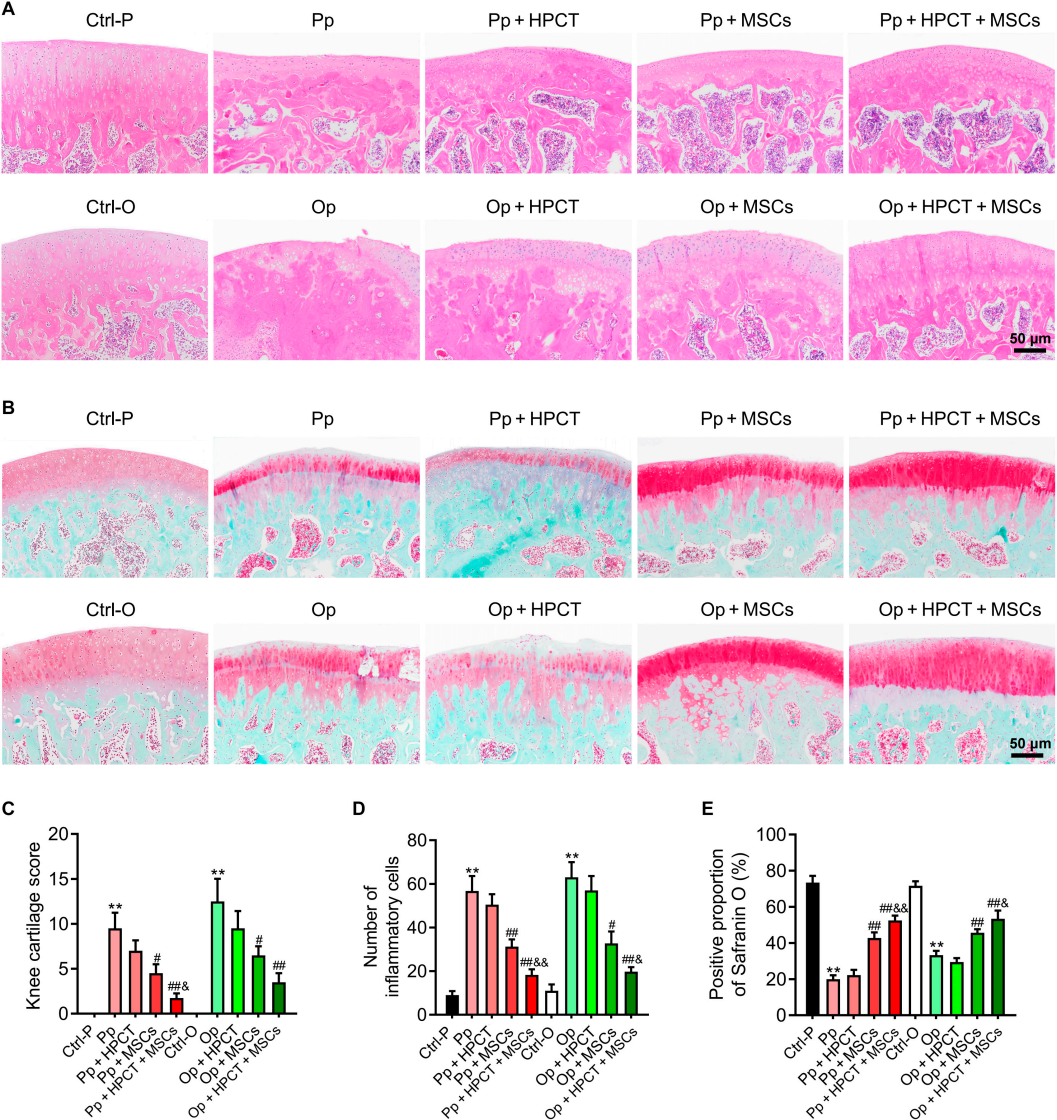
HPCT hydrogel/MSC therapy maintains joint tissue homeostasis 1 month after treatment cessation in operative- and papain-induced OA model. (A and B) Representative images of H&E staining (A) and Safranin O/Fast Green staining (B) from rat cartilage 1 month after HPCT hydrogel/MSC therapy. (C) Quantitative scoring of articular cartilage 1 month after HPCT hydrogel/MSC therapy. (D) Quantitative analysis of inflammatory cell numbers in articular cartilage 1 month after HPCT hydrogel/MSC therapy. (E) Ratio of cartilage proteoglycan to bone tissue 1 month after HPCT hydrogel/MSC therapy. *n* = 8 per group. **P < 0.05, *P < 0.01, #*P* < 0.05, ##*P* < 0.01, &*P* < 0.05, &&*P* < 0.01.

To ensure that the HPCT hydrogel/MSC therapy could potentially serve as curative treatment for OA, we assessed cartilage status 1 month after treatment withdrawal. H&E staining revealed that long-term OA modeling resulted in decreased cartilage surface flatness and significant inflammatory cell infiltration. In contrast, the HPCT hydrogel/MSCs group exhibited restored cartilage surface smoothness and minimal inflammatory infiltration (Fig. 7A to D). Safranin O/Fast Green staining showed a significant increase in the cartilage glycosaminoglycan-to-bone ratio in the combined therapy group similar to the untreated group, indicating sustained cartilage regeneration (Fig. 7B and E).

These findings demonstrate that the long-term administration of HPCT hydrogel combined with MSC therapy effectively reverses cartilage damage, mitigates inflammation, enhances joint morphology, and promotes sustained cartilage regeneration with an excellent safety profile.

## Discussion

Despite remarkable progress in deciphering the immunomodulation efficacy of MSC therapy in OA treatment, it has long been constrained by the limited retention time and compromised functionality of transplanted MSCs [31]. Here, we demonstrate a chitin-based thermosensitive hydrogel that addresses these challenges by enhancing MSCs’ self-renewal and anti-inflammatory capacity. The finding that MSCs embedded within the HPCT hydrogel exhibit superior biological activity suggests that HPCT hydrogel may play a role in maintaining MSC identity. Indeed, we observed elevated expression of stemness markers such as CD44, CD90, and CD105, highlighting the role of the 3D microenvironment in preserving MSC identity [32]. We propose that the spatial architecture promotes critical intracellular signaling pathways and gene expression patterns essential for maintaining MSC identity and functionality. The incorporation of chitin derivatives, specifically NAG and CTOS, further enhances the regenerative potential of MSCs by up-regulating anti-inflammatory genes and stimulating type II collagen synthesis to promote MSC self-renewal. These effects align with the activation of the Nrf2/GPX4 antioxidant pathway, reducing pro-inflammatory cytokines such as IL-6 and IL-8 while protecting cells from oxidative stress [33].

Our RNA-seq analysis highlights the involvement of HPCT hydrogels in regulating tight junctions, ECM dynamics, and the Hippo signaling pathway, all of which are pivotal to cell mechanics and fate determination. ECM properties—such as stiffness, viscoelasticity, and deformation—play a significant role in shaping cellular responses. Increased ECM stiffness enhances mechanical feedback, leading to elevated cellular stress and the secretion of stress-related factors through YAP protein nuclear translocation [34]. Modulating ECM characteristics can influence YAP activity and thus regulate stem cell fate [35]. Indeed, our findings show that MSCs embedded in HPCT hydrogels exhibit decreased activation of the Hippo pathway and reduced expression of YAP. While conventional 3D culture up-regulates YAP expression, embedding MSCs within the hydrogel environment significantly down-regulated its levels. This is noteworthy because YAP expression typically increases in rigid environments, driving MSC differentiation into adipogenic or osteogenic lineages and amplifying inflammatory responses [36–38]. In contrast, softer environments, like those provided by HPCT hydrogels, suppress YAP expression, thereby enhancing MSC self-renewal, mitigating inflammation, and preventing apoptosis.

We show that the HPCT hydrogel directly mitigates chondrocyte ferroptosis, an emerging pathogenic mechanism in OA. Recent studies indicate that Piezo1 facilitates calcium entry and supports cell self-renewal and growth by suppressing the NOTCH signaling pathway or modulating Wnt signaling, yet its overactivation may lead to iron ion entry [30,39]. Our findings show that HPCT treatment is associated with reduced Piezo1 expression and enhanced GSH/GPX4 antioxidant capacity, coinciding with decreased ferroptotic responses under mechanical-stress conditions [40,41]. Importantly, although we observed reduced Piezo1 expression together with attenuated ferroptotic markers following HPCT treatment, our current data do not establish a direct causal relationship between Piezo1 regulation and ferroptosis suppression. In addition, Piezo1 is primarily regulated at the level of channel activity rather than transcription, and thus changes in protein expression may not fully reflect its functional state. Future studies incorporating direct assessment of Piezo1 activity, such as calcium influx assays or electrophysiological measurements, as well as genetic or pharmacological perturbation, will be required to clarify the mechanistic contribution of Piezo1 in this context. This phenotype has never been observed in OA treatments with hyaluronic acid, which can only lubricate the joint and relieve the pain. The presence of embedded MSCs within the hydrogel amplifies this protective effect, likely by secreting more anti-inflammatory and regenerative factors [42,43], which promote cartilage repair and inflammation relief via paracrine signaling, emphasizing the dual role of MSCs in modulating both cellular and molecular pathways critical for OA management.

Our bioluminescent imaging data show that HPCT hydrogel is capable of extending MSC retention at the injection site. The hydrogel’s unique pore structure provides physical protection against immune cell attacks from macrophages and natural killer cells. This shielding effect prolonged MSC retention from 5 d (without HPCT hydrogel) to more than 7 d (with HPCT hydrogel), ensuring sustained therapeutic activity. Furthermore, MSCs embedded in the hydrogel exhibited potent immunomodulatory effects by inhibiting pro-inflammatory cytokines such as IL-1β and TNF-α, while promoting anti-inflammatory factors such as IL-10 and TSG-6. Several studies indicate that IL-10 and TSG-6 can suppress the TLR2/NF-κB pathway, which in turn decreases MMP13 expression and inflammatory factor release [44,45]. However, we did not observe a change in the TGF-β1 signaling. We propose that the homeostatic balance of the TGF-β signaling pathway is crucial for cartilage repair, as it has been implicated in modulating NF-κB Interacting LncRNA expression, which may contribute to anti-inflammatory effects and joint repair. Conversely, dysregulated TGF-β signaling has been linked to increased MMP13 transcription, reduced Collagen type II levels, and cartilage degradation [46,47]. These properties alleviate joint inflammation and create a conducive environment for cartilage repair. We propose that, in a normal microenvironment, TGF-β signaling may promote cartilage differentiation and inhibit inflammation, whereas in an inflammatory context, it may attract inflammatory cells, leading to synovial hyperplasia and fibrosis. Indeed, our results suggest that low levels of TGF-β1 may be more conducive to OA recovery, while high concentrations may exacerbate disease progression. Alternatively, the absence of detectable changes in joint cavity TGF-β1 levels could be due to its consumption during cartilage repair or MSC differentiation.

Lastly, the HPCT hydrogel’s injectability and phase-transition properties facilitate minimally invasive administration, reducing patient burden compared to invasive operative techniques [48]. Long-term safety evaluations revealed no systemic immune responses or adverse effects, even after repeated treatment cycles, underscoring the clinical feasibility and scalability of this approach. Autologous MSC transplantation, derived from bone marrow, adipose tissue, or umbilical cord, further addresses immune rejection and ethical concerns, further enhancing the translational potential of this therapy. Nonetheless, the variability in MSC characteristics among patients poses a challenge for consistent clinical outcomes. Patient-induced pluripotent-stem-cell-derived MSCs, which can be engineered for high functionality and uniform quality, represent a promising avenue for future research [49,50]. Additionally, combining HPCT hydrogel with advanced gene editing of MSCs and integrating potential drugs to enhance immune modulation and anti-ferroptotic effects, promote MSC self-renewal, and direct differentiation could further augment therapeutic efficacy.

This work presents a bioactive, thermosensitive HPCT hydrogel that substantially enhances the therapeutic efficacy of MSC-based treatment for osteoarthritis. By providing a mechanically protective and biologically supportive microenvironment, the HPCT hydrogel improves MSC retention, preserves stem cell functionality, and amplifies their anti-inflammatory and regenerative effects. Mechanistically, this system is associated with modulation of mechanotransduction-related pathways, including the Piezo1–GPX4 axis, which may contribute to reduced ferroptotic responses. The combined HPCT/MSC therapy promotes sustained cartilage repair, restores joint homeostasis, and demonstrates excellent biocompatibility in both short- and long-term evaluations. These findings position HPCT-based hydrogel delivery as a promising and translatable strategy for improving stem cell therapies in degenerative joint diseases.

## Data Availability

The data that support the findings of this study are available from the corresponding author upon reasonable request.
