# Supplementary material for: Bioactive Chitin-Based Thermosensitive Hydrogel Reinforces Stem Cell Therapy for Osteoarthritis
Source: Biomater Res. 2026 Jun 23;30:0382. doi: 10.34133/bmr.0382 (PMC13287448; doi:10.34133/bmr.0382)
Supplement: Supplementary 1 — Figs. S1 to S12 [file bmr.0382.f1.zip › Supplementary Materials.docx]

**Figure S1. Establishment of HPCT hydrogel/MSCs *in vitro* culture system.** (A) Molecular structure of HPCT. (B) Representative H&E staining images of MSC morphology after embedded within HPCT hydrogel. (C) Morphology of MSCs cultured in HPCT hydrogel with or without ROCK inhibitor. (D) Morphology of MSCs after re-embedding in HPCT hydrogel. (E) Morphology of MSCs cultured under stationary versus shaking conditions. (F) Quantification of MSCs in HPCT hydrogel with or without ROCK inhibitor. (G) Quantification of MSCs cultured under stationary versus shaking conditions. (H–K) Flow cytometry analysis of MSC surface markers after embedded within HPCT hydrogel, including representative images of CD44^+^, CD90^+^, and CD105^+^ populations (H) and corresponding quantification (I–K).**p* < 0.05, ***p* < 0.01, #*p* < 0.05, ##*p* < 0.01.

**Figure S2. Gene Ontology analyses on genes up/downregulated in across 2D, conventional 3D, and hydrogel-embedded culture systems.** (A) Gene Ontology analysis on genes upregulated across 2D, conventional 3D, and hydrogel-embedded culture systems.。(B) Gene Ontology analysis on genes downregulated across 2D, conventional 3D, and hydrogel-embedded culture systems. (C) KEGG analysis on genes downregulated across 2D, conventional 3D, and hydrogel-embedded culture. (D-I) Genomic coverage data tracks for RNA-seq experiments in MSCs in 2D culture and embedded in HPCT hydrogel at negative regulation of T cell proliferation(D) and apoptotic process(F), inflammatory response(E), actin cytoskeleton organization(G), cell-matrix adhesion(H) and extracellular matrix organization(I) targets.

**Figure S3.** **Effects of matrix stiffness on MSC stemness and YAP signaling.** (A-D) qPCR analysis of gene expression of YAP (A), CD44 (B), CD90 (C), and CD105 (D) in MSCs cultured under 1.5% and 4% HPCT conditions.

**Figure S4. HPCT degradation products on MSCs self-renewal.** (A) HPGPC analysis of HPCT degradation products in synovial fluid at day 0 and day 9 post-injection. (B-C) CCK8 analysis (B), and morphology (C) of MSCs in the presence of GS, NAG, CTOS, and CSOS. (D-I) qPCR analysis of IL-10 (D), TGF-β1 (E), TSG-6 (F), CD44 (G), CD90 (H), and CD105 (I) in MSCs with NAG or CTOS. **p* < 0.05, ***p* < 0.01, #*p* < 0.05, ##*p* < 0.01.

**Figure S5. HPCT hydrogel on inflammatory response in cartilage and muscle tissues.** (A-B) Representative HE staining images (A), and quantification of inflammatory cell numbers (B) of cartilage on days 9, 18, and 36 following HPCT hydrogel injection. (C-D) Representative HE staining images (C) and quantification of inflammatory cell numbers (D) of muscle tissue on days 9, 18, and 36. Scale bar: 50 μm. n = 8 per group. **p* < 0.05, ***p* < 0.01, #*p* < 0.05, ##*p* < 0.01.

**Figure S6. MSCs retention in joint cavity of operative- and papain-induced OA model.** (A) Schematic representation of operative- and papain-induced OA model. (B) Quantification of fluorescence signals over time in MSCs within the joint cavity using Living Image software. n = 8 per group. **p* < 0.05, ***p* < 0.01, #*p* < 0.05, ##*p* < 0.01.

**Figure S7. HPCT hydrogel/MSCs therapy 9-day treatment on cartilage repair.** (A-G) Quantification of ICRS scores (A), gross morphology scores (B), histopathological scores (C), inflammatory cell numbers (D), cartilage proteoglycan-to-bone ratio (E), percentage of collagen II-positive cells (F), and MMP13 fluorescence expression levels (G) in cartilage tissues. n = 8 per group. **p* < 0.05, ***p* < 0.01, #*p* < 0.05, ##*p* < 0.01, &*p* < 0.05, &&*p* < 0.01.

**Figure S8. Short-term *in vivo* safety evaluation of HPCT hydrogel/MSCs therapy.** (A-F) Hematological analysis of RBC (A), WBC (B), PLT (C), HGB (D), Lymph (E), and Gran (F) levels in each group. (G-J) Biochemical analysis of ALT (G), AST (H), ALP (I), and ALB (J) levels. (K-L) Spleen index (K) and thymus index (L) in each group. n = 8 per group. **p* < 0.05, ***p* < 0.01, #*p* < 0.05, ##*p* < 0.01, &*p* < 0.05, &&*p* < 0.01.

**Figure S9. HPCT hydrogel/MSCs therapy on inflammation and ferroptosis.** (A–B) Effects of HPCT/MSCs treatment on chondrocyte proliferation under inflammatory conditions, including representative images (A) and quantification (B). (C–E) Effects of HPCT/MSCs treatment on TLR2/P65 signaling in chondrocytes under inflammatory conditions, including representative images (C) and quantitative analysis (D–E). (F–I) Quantification of TLR2 (F), P65 (G), GPX4 (H), and ROS (I) levels in cartilage tissues using ImageJ. (J–K) Biochemical analysis of lipid peroxidation markers LPO (J) and MDA (K) in synovial fluid at day 9. (L–M) Quantification of GPX4 (L) and Piezo1 (M) protein expression in chondrocytes following Yoda1-induced ferroptosis. **p* < 0.05, ***p* < 0.01, #*p* < 0.05, ##*p* < 0.01, &*p* < 0.05, &&*p* < 0.01.

**Figure S10. Long-term HPCT hydrogel/MSCs therapy on cartilage repair. (A) Schematic diagram of the long-term administration regimen.** (B) Quantification of luciferase signals in MSCs within the joint cavity on day 4 after each treatment session using Living Image software. (C-G) Quantification of ICRS scores (C), total cartilage scores (D), histopathological scores (E), inflammatory cell numbers (F), and cartilage proteoglycan-to-bone ratio (G) after 36 days of long-term treatment. n = 8 per group. **p* < 0.05, ***p* < 0.01, #*p* < 0.05, ##*p* < 0.01, &*p* < 0.05, &&*p* < 0.01.

**Figure S11. HPCT hydrogel/MSCs therapy on the levels of secreted factors in long-term treatment.** (A-E) ELISA analysis showing the levels of IL-10 (A), TSG-6 (B), TGF-β1 (C), IL-1β (D), TNF-α (E), and biochemical analysis of GSH (F) in synovial fluid after 36 days of treatment. n = 8 per group. **p* < 0.05, ***p* < 0.01, #*p* < 0.05, ##*p* < 0.01, &*p* < 0.05, &&*p* < 0.01.

**Figure S12. Long-term *in vivo* safety evaluation of HPCT hydrogel/MSCs therapy.** (A-F) Hematological analysis of RBC (A), WBC (B), PLT (C), HGB (D), Lymph (E), and Gran (F) levels. (G-J) Biochemical analysis of ALT (G), AST (H), ALP (I), and ALB (J) levels. (K-L) Spleen index (K) and thymus index (L) in each group. n = 8 per group. **p* < 0.05, ***p* < 0.01, #*p* < 0.05, ##*p* < 0.01, &*p* < 0.05, &&*p* < 0.01.
